# Association of dietary inflammatory index with liver fibrosis and fatty liver index in a population with metabolic dysfunction-associated steatotic liver disease: A cross-sectional study

**DOI:** 10.3389/fnut.2025.1594192

**Published:** 2025-06-24

**Authors:** Ziqi Sang, Han Wang, Yan Leng, Xupeng Huang, Peng Sun, Ruolin Wang, Tiejun Liu, Houbo Deng

**Affiliations:** ^1^College of Traditional Chinese Medicine, Changchun University of Chinese Medicine, Changchun, China; ^2^Institute of Chinese Materia Medica, China Academy of Chinese Medical Sciences, Beijing, China; ^3^Department of Hepatology, The First Affiliated Hospital of Changchun University of Traditional Chinese Medicine, Changchun, China

**Keywords:** MASLD, dietary inflammatory index, hepatic fibrosis, Fatty liver index, systemic immune-inflammation index (SII)

## Abstract

**Background:**

Hepatic fibrosis and the fatty liver index (FLI) are critical indicators for assessing the progression and severity of metabolic dysfunction-associated steatotic liver disease (MASLD) and serve as valuable reference markers for predicting MASLD-related risks. The Dietary Inflammation Index (DII) quantifies the inflammatory effects of dietary intake and has been extensively utilized in nutritional and epidemiological studies. Although studies have been conducted to confirm the correlation between dietary quality and MASLD in the general population, this study sought to further explore the association between the DII and key indicators of liver disease severity—namely hepatic fibrosis and the FLI—within a cohort diagnosed with MASLD. In addition, the potential mediating role of the systemic immune inflammatory index (SII) in these associations was also investigated.

**Methods:**

This cross-sectional study was based on data from the 2017–2020 cycles of the U.S. National Health and Nutrition Examination Survey (NHANES), a nationally representative program designed to assess the health and nutritional status of the population. In this study, we analyzed the correlation of DII with FLI and liver fibrosis in a population of patients with MASLD by linear regression, logistic regression, RCS curves and subgroup analysis. A mediation model was applied to assess the potential intermediary effect of SII on the associations between DII, FLI, and hepatic fibrosis.

**Results:**

The results of this study indicate that, after adjusting for all covariates, the Dietary Inflammatory Index (DII) was not significantly associated with the Fatty Liver Index (FLI) among U.S. adults with MASLD (β = 0.32, 95% CI: −1.393 to 2.034, *P* = 0.631). Similarly, no significant association was observed between DII and the risk of liver fibrosis (OR = 1.152, 95% CI: 0.885 to 1.499, *P* = 0.210). Subgroup analyses further demonstrated that these associations were not modified by demographic or metabolic stratification variables, and the relationship appeared to be nonlinear.

**Conclusion:**

In U.S. adults with MASLD, no significant association was found between DII and the risk of liver fibrosis or elevated FLI. Although DII is linked to various chronic diseases, its role in MASLD appears limited and non-specific, particularly in capturing intermittent disease progression. No mediating effect of SII was observed. These findings underscore the importance of carefully considering dietary factors in the clinical evaluation of MASLD progression. The potential relationship between diet and liver disease progression warrants further investigation.

## 1 Introduction

Metabolic dysfunction-associated steatotic liver disease (MASLD) redefines the classification of steatotic liver disease (SLD), focusing on the interplay between hepatic steatosis and metabolic dysfunction. Replacing non-alcoholic fatty liver disease (NAFLD), MASLD leverages multi-omics approaches for more precise stratification than metabolic dysfunction-associated fatty liver disease (MAFLD) and helps reduce disease-related stigma (1, 2). It represents a progressive pathological spectrum beginning with isolated steatosis and potentially advancing to MASH, cirrhosis, and ultimately hepatocellular carcinoma. MASLD is closely associated with type 2 diabetes, atherosclerosis, and hypertension, significantly increasing all-cause mortality risk (3, 4). Globally, it affects ~1.27 billion individuals, with higher prevalence in males (51.41%). In type 2 diabetes patients, prevalence reaches 65.3%, and exceeds 70% in Eastern Europe and the Middle East. Based on age-standardized incidence rates (ASIR), new MASLD cases are projected to reach 667.58 million by 2045 (5, 6).

Liver fibrosis and the fatty liver index (FLI) are important indicators of disease progression and severity in MASLD. Growing evidence suggests that systemic inflammation plays a central role in promoting hepatic stellate cell activation, extracellular matrix deposition, and lipid metabolism dysregulation, thereby accelerating liver fibrosis and steatosis. Consequently, systemic inflammation has predictive value in assessing the risk of disease progression, and controlling inflammatory burden has become a key strategy to slow down MASLD development (1, 7, 8).

Modifying lifestyle habits, especially dietary interventions, has been shown to significantly lower MASLD prevalence in the general population, highlighting its role as a crucial preventive strategy (9–12). By providing a numerical estimation of dietary-induced inflammation, the DII has been broadly employed in epidemiological research to examine its relationship with systemic inflammation and various chronic diseases, such as cancer, diabetes, and cardiovascular conditions (13, 14). Recent studies have demonstrated that higher DII values are closely linked to fatty liver risk, especially in obese populations, emphasizing the contribution of inflammatory dietary patterns to hepatic disease development (15). Further findings by Ma et al. (16) indicated that individuals with elevated dietary inflammatory load exhibit a higher likelihood of developing metabolic syndrome and face increased all-cause and cardiovascular mortality risks. In contrast, adherence to an anti-inflammatory dietary pattern is associated with reduced systemic inflammation (17, 18). Thus, dietary interventions not only offer a cost-effective approach for MASLD prevention and management but may also delay disease progression by modulating inflammatory processes (18–22).

The systemic immune-inflammation index (SII), derived from neutrophil, lymphocyte, and platelet counts, has emerged as a composite marker for evaluating systemic inflammatory status. Strongly associated with metabolic dysfunction, liver fibrosis, and adverse cardiovascular outcomes, SII provides a more accurate reflection of systemic immune status than conventional inflammatory markers and offers important insights into MASLD progression (23).

Although previous studies have established a link between dietary indices and MASLD in the general population (24–26), their role in disease progression remains unclear. In particular, the associations between DII and progression markers such as liver fibrosis and the Fatty Liver Index (FLI) are not well defined. Whether systemic inflammation, as reflected by the systemic immune-inflammation index (SII), mediates these associations also remains unknown. Accordingly, this study investigates the relationship between the Dietary Inflammatory Index (DII) and liver fibrosis and FLI in individuals with MASLD, while further examining whether the systemic immune-inflammation index (SII) mediates these associations, aiming to elucidate inflammatory pathways involved in MASLD progression.

## 2 Materials and methods

### 2.1 Study population

**Figure 1:**
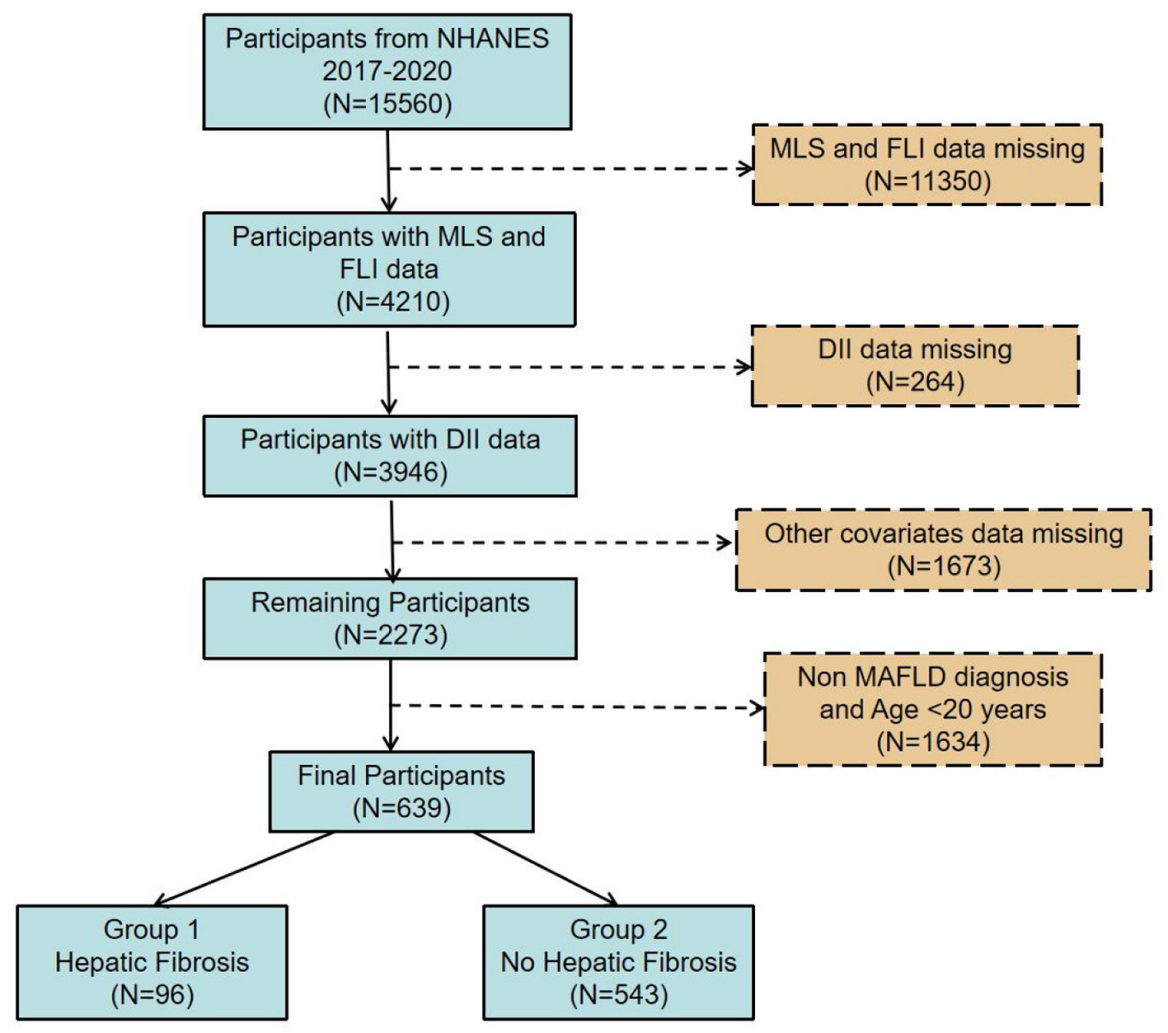
Flow diagram illustrating the participant selection process from the 2017–2020 NHANES dataset.

Based on data from the 2017–2020 NHANES cycles, this cross-sectional analysis comprised 15,560 individuals who met the criteria for complete data inclusion. After excluding missing values for liver fibrosis (mLS) and FLI, 4,210 cases remained. Further exclusions for missing DII and other covariates left 3,946 and 2,273 cases, respectively. After removing non-MAFLD individuals and those under 20, 639 cases were included, with 96 positive for liver fibrosis (Figure 1).

The pre-pandemic 2017–2020 cycle includes data from the 2017–2018 and 2019–2020 survey periods. Due to the COVID-19 outbreak, some field operations during the 2019–2020 cycle were suspended. Nonetheless, the dataset continues to reflect a nationally representative sample of the non-institutionalized U.S. civilian population during the approximately 3.2-year period preceding the COVID-19 pandemic.

### 2.2 Measurements

#### 2.2.1 Criteria for the diagnosis of metabolic dysfunction-associated steatotic liver disease

The diagnostic criteria for MASLD require the presence of hepatic steatosis, which can be identified through histological examination (liver biopsy), imaging techniques, or blood biomarkers, in combination with at least one of the following five metabolic risk abnormalities (1): Overweight or obesity, defined as a body mass index (BMI) ≥ 25 kg/m^2^, or increased waist circumference ≥ 94 cm in men and ≥ 80 cm in women of Caucasian descent, with race-specific thresholds applied where appropriate; type 2 diabetes or impaired glucose metabolism (prediabetes defined by FPG 100–125 mg/dL, 2-h post-load glucose 140–199 mg/dL, or HbA1c 5.7%−6.4%); hypertension (≥130/85 mmHg or use of antihypertensive medication); elevated triglycerides (≥150 mg/dL or lipid-lowering treatment), low HDL-C (<40 mg/dL in men, <50 mg/dL in women or lipid-lowering treatment).

The presence and extent of hepatic steatosis were determined using values obtained from the controlled attenuation parameter (CAP) technique, with the threshold set at 248 dB/m based on findings from large meta-analyses, together with the latest consensus on the diagnosis of MASLD (27–29). Participants with CAP values below 248 dB/m were categorized as non-MAFLD and excluded from the study.

#### 2.2.2 Determination of liver fibrosis and fatty liver indices

Transient elastography (TE) is a widely used, FDA-approved non-invasive technique for assessing liver stiffness and fibrosis (27–30). Transient elastography was performed at the NHANES Mobile Examination Center using the FibroScan^®^ 502 V2 Touch, incorporating M and XL probes to measure liver stiffness. Eligibility for liver stiffness analysis required a minimum 3-hour fasting period, at least 10 valid measurements, and an interquartile range-to-median ratio of <30%. Multiple meta-analyses have assessed and established optimal liver stiffness thresholds for diagnosing various stages of fibrosis (26, 31). In this study, significant fibrosis (F2) was defined using a median liver stiffness cutoff of 8.0 kPa (27, 32). Participants with MAFLD but without significant fibrosis were classified as controls.

The Fatty Liver Index (FLI) is a validated, non-invasive tool used to estimate the likelihood of hepatic steatosis. It predicts the likelihood of fatty liver using basic clinical and laboratory indicators, such as triglyceride levels, body mass index (BMI), γ-glutamyl transferase (GGT), and waist circumference. According to the FLI assessment model proposed by Jung et al. (33) the FLI is computed using the following formula:


(1) $$\begin{array}{l} FLI=\\ \frac{\left(e\left[0.953\times \text{ln}\left(\text{TG}\right)+0.139\times \text{BMI}+0.718\times \text{ln}\left(\text{GGT}\right)+0.053\times \text{WC}-15.745\right)\right]}{\left(1+e\left[0.953\times \text{ln}\left(\text{TG}\right)+0.139\times \text{BMI}+0.718\times \text{ln}\left(\text{GGT}\right)+0.053\times \text{WC}-15.745\right]\right)}\\ \times 100\; \end{array}$$

where In is the natural logarithm, e is the base of the natural logarithm, TG is in mmol/L, GGT is in U/L, WC is in cm, and scores range from 0 to 100.

#### 2.2.3 Assessment and calculation of the dietary inflammatory index

This study utilized NHANES dietary intake data, obtained from two 24-h dietary recall interviews, to estimate energy and nutrient consumption. Dietary intake data from the first 24-h recall were utilized as the main reference for analysis. The Dietary Inflammatory Index (DII), proposed by Shivappa et al., provides a validated approach to evaluating the inflammation-related potential of diet through nutrient composition analysis. While the DII incorporates 45 nutrients, it remains valid with a minimum of 30. In this study, 27 nutrients were included, covering macronutrients (energy, fat, saturated fat, monounsaturated fat, polyunsaturated fat, protein, carbohydrates, fiber, and alcohol), specific fatty acids (ALA, EPA, DHA, DPA, linoleic acid, and arachidonic acid), micronutrients (cholesterol, niacin, vitamins A, B1, B2, B6, B12, C, D, and E, iron, zinc, selenium, magnesium, folate, and β-carotene), and caffeine. The total n-3 fatty acid intake was calculated by summing ALA, EPA, DHA, and DPA, while n-6 fatty acid intake was determined by adding linoleic and arachidonic acids (13, 34).

To ensure consistency in nutrient intake measurements, the global dietary standards database was used to derive the mean and variability of each nutrient, allowing raw values to be transformed into Z-scores. The Z-scores were converted to percentiles, then scaled by a factor of two and reduced by one to yield a symmetric distribution centered at zero. The DII score ranges from −1, indicating the highest anti-inflammatory potential, to +1, representing the strongest pro-inflammatory effect. It is calculated by assigning weights to each nutrient based on its inflammatory impact and summing the values. DII scores below zero represent anti-inflammatory dietary intake, whereas scores above zero are indicative of pro-inflammatory dietary characteristics (35–38).

### 2.3 Covariates assessment

The selection of covariates was guided by prior studies and clinical significance, incorporating data from demographics, physical examinations, laboratory tests, and questionnaires. Standardized household interviews were used to collect demographic information, including age, self-reported gender, race/ethnicity, and educational attainment. Lifestyle factors were assessed, with alcohol consumption and smoking statuscategorized into currently smoking or drinking alcohol, and not currently. Anthropometric and clinical measurements included body mass index (BMI), calculated as weight (kg) divided by height squared (m^2^). SII was computed based on complete blood count data, using the following equation: SII = platelet count multiplied by neutrophil count, divided by lymphocyte count. Hypertension was defined by a physician-diagnosed history or at least three separate non-consecutive measurements of systolic blood pressure ≥140 mmHg or diastolic blood pressure ≥90 mmHg within 1 year of baseline assessment. Diabetes and hyperlipidemia were identified based on self-reported medical history. Laboratory data, including total cholesterol and parameters for FLI calculation, were extracted from NHANES datasets.

### 2.4 Statistical methods

To evaluate baseline differences, one-way ANOVA was applied for continuous variables and chi-square tests for categorical variables. The associations between the Dietary Inflammatory Index (DII), liver fibrosis, and the Fatty Liver Index (FLI) in individuals with MASLD were examined using multivariable linear and logistic regression models. In both regression analyses, Model 1 was unadjusted; Model 2 adjusted for age, sex, race, education level, marital status, and the ratio of family income to poverty; and Model 3 included further adjustments for BMI, smoking status, alcohol consumption, diabetes, hyperlipidemia, hypertension, white blood cell count, platelet count, SII, and hs-CRP. Additionally, mediation analysis was conducted to explore the intermediary role of SII in the relationships among DII, FLI, and hepatic fibrosis, with estimation of the proportion of the effect mediated through SII.

To explore potential nonlinear dose–response relationships between DII, liver fibrosis, and FLI, restricted cubic spline (RCS) models were applied. Additionally, threshold effect analysis was conducted to identify inflection points and evaluate the presence of nonlinearity. In addition, stratified analyses were conducted according to sex, age group, body mass index (BMI), and the presence of hypertension, hyperlipidemia, and diabetes, in order to evaluate whether these factors modify the associations between DII, hepatic fibrosis, and FLI. All analyses were conducted with consideration of NHANES' complex multistage sampling framework, and statistical significance was defined as *p* < 0.05.

## 3 Results

### 3.1 Characteristics of participants

Table 1 presents the demographic and clinical characteristics of NHANES MASLD participants aged 20 and older from the 2017–2020 dataset, stratified by gender. Among the 639 subjects, the mean (SE) age was 52.76 years, and the mean (SE) DII was 1.29. Among them, 13.87% of participants had symptoms of liver fibrosis. Baseline data, after weighted ANOVA and chi-square tests, showed that women and men differed in BMI (33.66 vs. 31.20 kg/m^2^), DII (1.74 vs. 0.98), Platelet (254.76 vs. 225.70 × 10^9^/L), SII (547.58 vs. 483.38), hs-CRP(4.72 vs. 2.86 mg/L) and Ratio Of family income to poverty(3.22 vs. 3.62), the above differences are statistically significant (*P* < 0.05). Additionally, females demonstrated a higher prevalence of hyperlipidemia (87.99% vs. 73.85%) and a lower rate of alcohol consumption (77.97% vs. 91.13%) compared to males. Significant gender differences were also observed in marital status and racial/ethnic distribution (*P* = 0.01 and *P* = 0.04, respectively). In contrast, no statistically significant Gender-based differences were found in age, Fatty Liver Index (FLI), white blood cell count, educational attainment, diabetes prevalence, smoking status, or hypertension. These findings suggest that although men and women exhibit broadly similar metabolic risk profiles, notable disparities exist in inflammatory markers and lifestyle behaviors.

**Table 1:** Baseline characteristics of study participants.

**Characteristics**	**Total**	**Gender**		***P* value**
		**Female**	**Male**	
Age (years)	52.76 (50.75, 54.76)	54.89 (51.88, 57.91)	51.27 (48.91, 53.64)	0.06
BMI, kg/m^2^	32.21 (31.52, 32.90)	33.66 (32.81, 34.52)	31.20 (30.27, 32.12)	< 0.001
DII	1.29 (1.06, 1.52)	1.74 (1.46, 2.01)	0.98 (0.68, 1.29)	< 0.001
FLI	69.14 (66.14, 72.14)	68.91 (65.40, 72.42)	69.29 (65.50, 73.08)	0.86
Wbc	6.89 (6.64, 7.14)	6.93 (6.63, 7.22)	6.86(6.56, 7.16)	0.71
Platelet	237.59 (230.85, 244.34)	254.76 (245.27, 264.25)	225.70 (218.67, 232.72)	< 0.0001
SII	509.66 (470.60, 548.72)	547.58 (497.66, 597.49)	483.38 (439.00, 527.76)	0.03
hs-CRP	3.62 (3.09, 4.16)	4.72 (3.87, 5.58)	2.86 (2.26, 3.45)	0.001
Ratio of family income to poverty	3.46 (3.22, 3.70)	3.22(2.91, 3.54)	3.62 (3.39, 3.86)	0.01
**Race/ethnicity**, ***n*** **(%)**				
Black	130 (7.39)	67 (10.18)	63 (5.46)	0.04
Mexican	82 (8.06)	43 (9.50)	39 (7.06)	
Other	179 (15.65)	91 (17.92)	88 (14.07)	
White	248 (68.90)	89 (62.40)	159 (73.41)	
**Education**				
9–11th Grade	58 (5.41)	27 (5.79)	31 (5.15)	0.22
College graduate or above	395 (66.99)	168 (62.22)	227 (70.29)	
High school Grad/GED or equivalent	144 (24.63)	71 (27.72)	73 (22.49)	
Less than 9th Grade	42 (2.97)	24 (4.27)	18 (2.08)	
**Marital**				
No	192 (23.28)	110 (30.68)	82 (18.15)	0.01
Yes	447 (76.72)	180 (69.32)	267 (81.85)	
**Diabetes**				
No	440 (76.60)	192 (74.29)	248 (78.20)	0.37
Yes	199 (23.40)	98 (25.71)	101(21.80)	
**Smoking status**, ***n*** **(%)**				
No	581 (93.28)	272 (93.25)	309 (93.30)	0.99
Yes	58 (6.72)	18 (6.75)	40 (6.70)	
**Alcohol user**				
No	121 (14.26)	85 (22.03)	36 (8.87)	0.004
Ye	518 (85.74)	205 (77.97)	313 (91.13)	
**Hypertension**				
No	280 (48.70)	124 (49.35)	156 (48.26)	0.85
Yes	359 (51.30)	166 (50.65)	193(51.74)	
**Hyperlipidemia**				
No	119 (20.36)	47 (12.01)	72 (26.15)	0.003
Yes	520 (79.64)	243 (87.99)	277 (73.85)	
**Hepatic fibrosis**				
No	543 (86.13)	243 (81.92)	300 (89.06)	0.15
Yes	96 (13.87)	47 (18.08)	49 (10.94)	

BMI: body mass index; DII, dietary inflammation index; FLI: fatty liver index; Wbc: white blood cell; SII hs-CRP: high sensitivity C-reactive protein.

### 3.2 Association analysis of DII with FLI, liver fibrosis and SII

Tables 2–4 summarize the results of univariate and multivariate linear and logistic regression analyses examining the associations between DII, FLI, liver fibrosis, and SII.

**Table 2:** Association between DII and FLI.

**Model**	**β**	**95% CI**	***P***
Model 1	1.544	(−0.375, 3.462)	0.110
Model 2	1.477	(−0.684, 3.637)	0.165
Model 3	0.32	(−1.393, 2.034)	0.631

Model 1: no covariates were adjusted. Model 2: age, gender, race, education level, marital and ratio of family income to poverty were adjusted. Model 3: age, gender, race, education level, marital, ratio of family income to poverty, BMI, smoking, alcohol use, wbc, platelet, SII, HS-CRP, hypertension, diabetes, and hyperlipidemia were adjusted.

As shown in Table 2, linear regression analysis yielded the following results: Model 1 (β = 1.544, 95% CI: −0.375, 3.462, *p* = 0.110), Model 2 (β = 1.477, 95% CI: −0.684, 3.637, *p* = 0.165), and Model 3 (β = 0.32, 95% CI: −1.393, 2.034, *p* = 0.631). These findings indicate no significant association between DII and FLI.

As shown in Table 3, logistic regression analysis yielded the following results: Model 1 (OR = 1.254, 95% CI: 1.058, 1.485, *p* = 0.011), Model 2 (OR = 1.232, 95% CI: 1.029, 1.474, *p* = 0.026), and Model 3 (OR = 1.152, 95% CI: 0.885, 1.499, *p* = 0.210). In Models 1 and 2, a positive association was observed between DII and liver fibrosis risk; however, this association lost statistical significance in Model 3 after full adjustment for covariates. As shown in Table 4, linear regression analysis yielded the following results: model 1 (β = 16.251, 95% CI: 2.994, 29.508, *p* = 0.018), Model 2 (β = 15.811, 95% CI: −2.149, 33.771, *p* = 0.080), and Model 3 (β = 12.028, 95% CI: −3.470, 27.525, *p* = 0.111). In Model 1 without adjusting for covariates, DII and SII showed a positive correlation, but no statistical significance was found after adjusting for other covariates.

**Table 3:** Association between DII and liver fibrosis.

**Model**	**OR**	**95% CI**	***P***
Model 1	1.254	(1.058, 1.485)	0.011
Model 2	1.232	(1.029, 1.474)	0.026
Model 3	1.152	(0.885, 1.499)	0.210

Model 1: no covariates were adjusted. Model 2: age, gender, race, education level, marital and ratio of family income to poverty were adjusted. Model 3: age, gender, race, education level, marital, ratio of family income to poverty, BMI, smoking, alcohol use, wbc, platelet, SII, HS-CRP, hypertension, diabetes, and hyperlipidemia were adjusted.

**Table 4:** Association between DII and SII.

**Model**	**β**	**95% CI**	***P***
Model 1	16.251	(2.994, 29.508)	0.018
Model 2	15.811	(−2.149, 33.771)	0.080
Model 3	12.028	(−3.470, 27.525)	0.111

Model 1: no covariates were adjusted. Model 2: age, gender, race, education level, marital and ratio of family income to poverty were adjusted. Model 3: age, gender, race, education level, marital, ratio of family income to poverty, BMI, smoking, alcohol use, hypertension, diabetes, and hyperlipidemia were adjusted.

### 3.3 Subgroup analysis

Tables 5, 6 present subgroup analyses stratified by gender, age, BMI, hypertension, hyperlipidemia, and diabetes to assess their influence on the relationship between DII, FLI, and liver fibrosis. We did not observe any interaction in Tables 5, 6. The findings suggest that sex, age, body mass index (BMI), as well as comorbidities such as hypertension, hyperlipidemia, and diabetes, did not significantly modify the associations between DII and either FLI or hepatic fibrosis among individuals with MASLD.

**Table 5:** Subgroup analysis of the relationship between DII and FLI.

**Character**	**Model 1**			**Model 2**			**Model 3**		
	**95% CI**	***p***	***p*** **for interaction**	**95% CI**	***p***	***p*** **for interaction**	**95% CI**	***p***	***p*** **for interaction**
Gender			0.189			0.149			0.053
Female	3.07 (0.68,5.46)	0.014		3.239 (0.50, 5.98)	0.024		2.154 (−0.03, 4.33)	0.052	
Male	0.577 (−2.34, 3.49)	0.686		0.733 (−2.24, 3.70)	0.605		−0.091 (−2.74, 2.56)	0.941	
Age			0.843			0.918			0.536
< 60	1.419 (−0.91, 3.75)	0.220		1.614 (−0.61, 3.84)	0.142		0.76 (−1.26, 2.78)	0.421	
≥60	1.728 (−1.11, 4.57)	0.220		1.455 (−1.40, 4.31)	0.291		0.132 (−2.91, 3.17)	0.924	
BMI			0.224			0.176			0.284
< 30	−0.969 (−3.19, 1.25)	0.376		−0.46 (−2.86, 1.93)	0.686		−0.544 (−3.12, 2.04)	0.645	
≥30	0.602 (−0.60, 1.80)	0.310		0.758 (−0.66, 2.17)	0.270		0.161 (−1.31, 1.64)	0.813	
Hyperten-Sion			0.983			0.963			0.85
Yes	1.604 (−0.88, 4.09)	0.195		2.013 (−0.78, 4.81)	0.145		1.256 (−1.73, 4.24)	0.370	
No	1.566 (−1.46, 4.59)	0.296		1.372 (−1.43, 4.17)	0.311		0.117 (−2.26, 2.49)	0.914	
Hyperlipi- Demia			0.165			0.209			0.03
No	3.332 (−0.27, 6.94)	0.068		3.525 (0.28, 6.77)	0.036		2.653 (−0.39, 5.70)	0.077	
Yes	0.859 (−1.06, 2.78)	0.364		0.919 (−1.38, 3.22)	0.405		0.03 (−2.12, 2.18)	0.975	
Diabetes			0.631			0.677			0.509
No	1.001 (−1.43, 3.43)	0.404		1.107 (−1.34, 3.55)	0.348		0.283 (−1.88, 2.45)	0.777	
Yes	1.896 (−0.89, 4.68)	0.173		2.212 (−0.70, 5.12)	0.125		2.219 (−0.63, 5.07)	0.113	

Model 1: no covariates were adjusted. Model 2: race, alcohol use, smoke, marital, education and ratio of family income to poverty were adjusted. Model 3: race, alcohol use, smoke, marital, education, ratio of family income to poverty, wbc, platelet, SII, hs-CRP were adjusted.

**Table 6:** Subgroup analysis of the relationship between DII and liver fibrosis.

**Character**	**Model 1**			**Model 2**			**Model 3**		
	**95% CI**	***p***	***p*** **for interaction**	**95% CI**	***p***	***p*** **for interaction**	**95% CI**	***p***	***p*** **for interaction**
Gender			0.798			0.739			0.617
Female	1.255 (0.91, 1.72)	0.152		1.361 (1.00, 1.85)	0.050		1.263 (0.90, 1.77)	0.152	
Male	1.182 (0.89,1.58)	0.244		1.118 (0.81, 1.55)	0.471		1.106 (0.79, 1.56)	0.529	
Age			0.548			0.565			0.394
< 60	1.298 (1.02, 1.65)	0.035		1.284 (0.10,1.66)	0.054		1.251 (0.93,1.68)	0.118	
≥60	1.188 (0.95,1.49)	0.125		1.168 (0.94, 1.46)	0.156		1.128 (0.88, 1.45)	0.307	
BMI			0.159			0.086			0.100
< 30	0.997 (0.75,1.32)	0.980		0.934 (0.70, 1.25)	0.615		0.923 (0.65, 1.32)	0.623	
≥30	1.276 (1.01, 1.61)	0.041		1.266 (1.02, 1.58)	0.036		1.215 (0.94,1.58)	0.129	
Hypertension			0.734			0.606			0.594
Yes	1.241 (0.97,1.58)	0.078		1.219 (0.94, 1.59)	0.132		1.178 (0.86, 1.61)	0.271	
No	1.324 (1.00, 1.75)	0.050		1.395 (0.97, 2.01)	0.072		1.323 (0.95, 1.84)	0.088	
Hyperlipidemia			0.339			0.288			0.226
No	1.552 (0.92, 2.63)	0.097		1.592 (0.84, 3.01)	0.134		1.485 (0.68, 3.22)	0.258	
Yes	1.204 (1.00, 1.45)	0.047		1.194 (1.01, 1.42)	0.042		1.141 (0.93,1.40)	0.185	
Diabetes			0.695			0.469			0.624
No	1.231 (0.97, 1.56)	0.085		1.234 (0.92, 1.66)	0.148		1.184 (0.86, 1.64)	0.269	
Yes	1.141 (0.86, 1.51)	0.342		1.163 (0.89, 1.52)	0.242		1.226 (0.88, 1.71)	0.204	

Model 1: no covariates were adjusted. Model 2: race, alcohol use, smoke, marital, education and ratio of family income to poverty were adjusted. Model 3: race, alcohol use, smoke, marital, education, ratio of family income to poverty, wbc, platelet, SII, hs-CRP were adjusted.

### 3.4 Dose-response relationship

**Figure 2:**
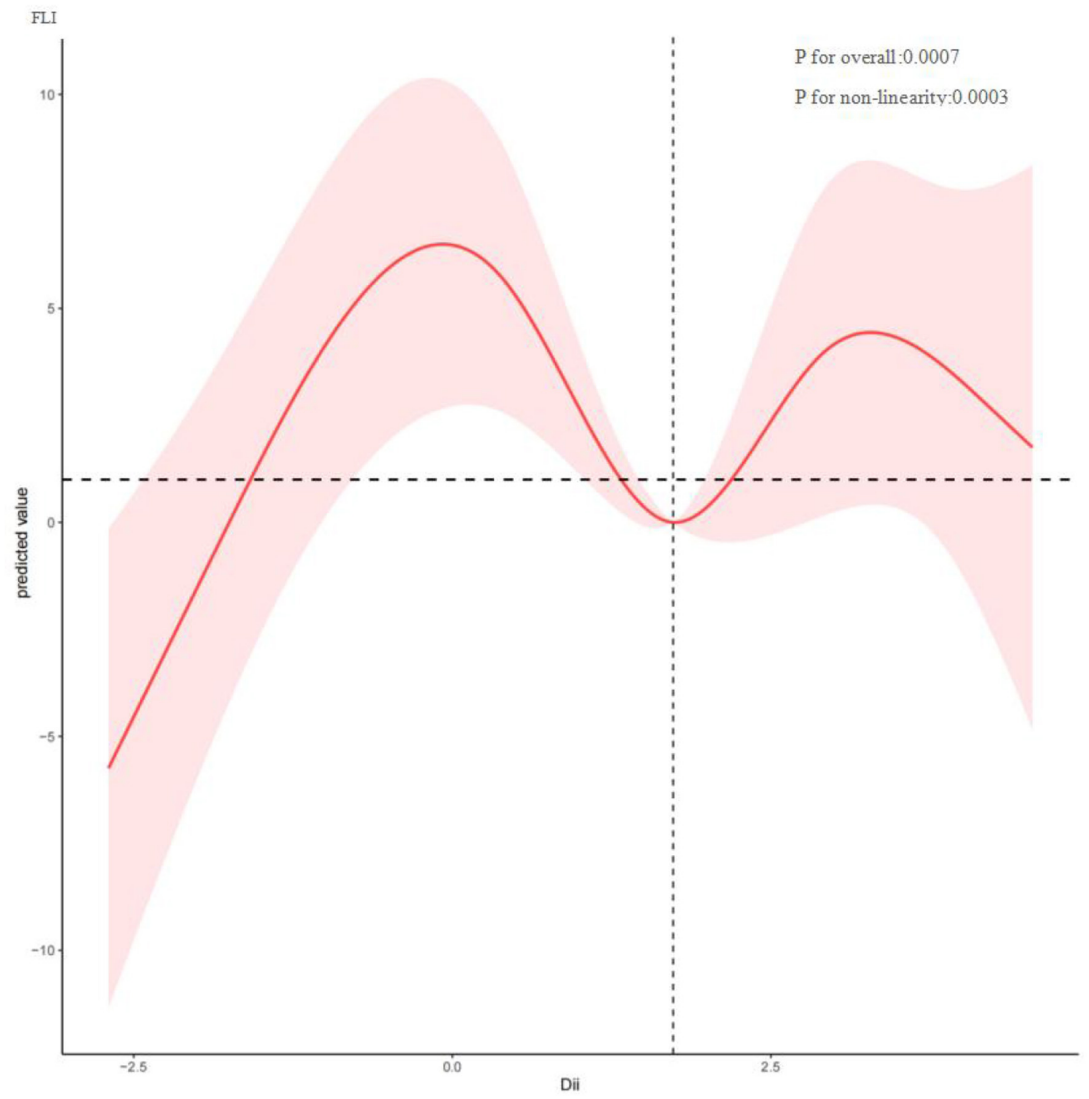
Restricted cubic spline curve (RCS) plot of the relationship between DII, and FLI.

**Figure 3:**
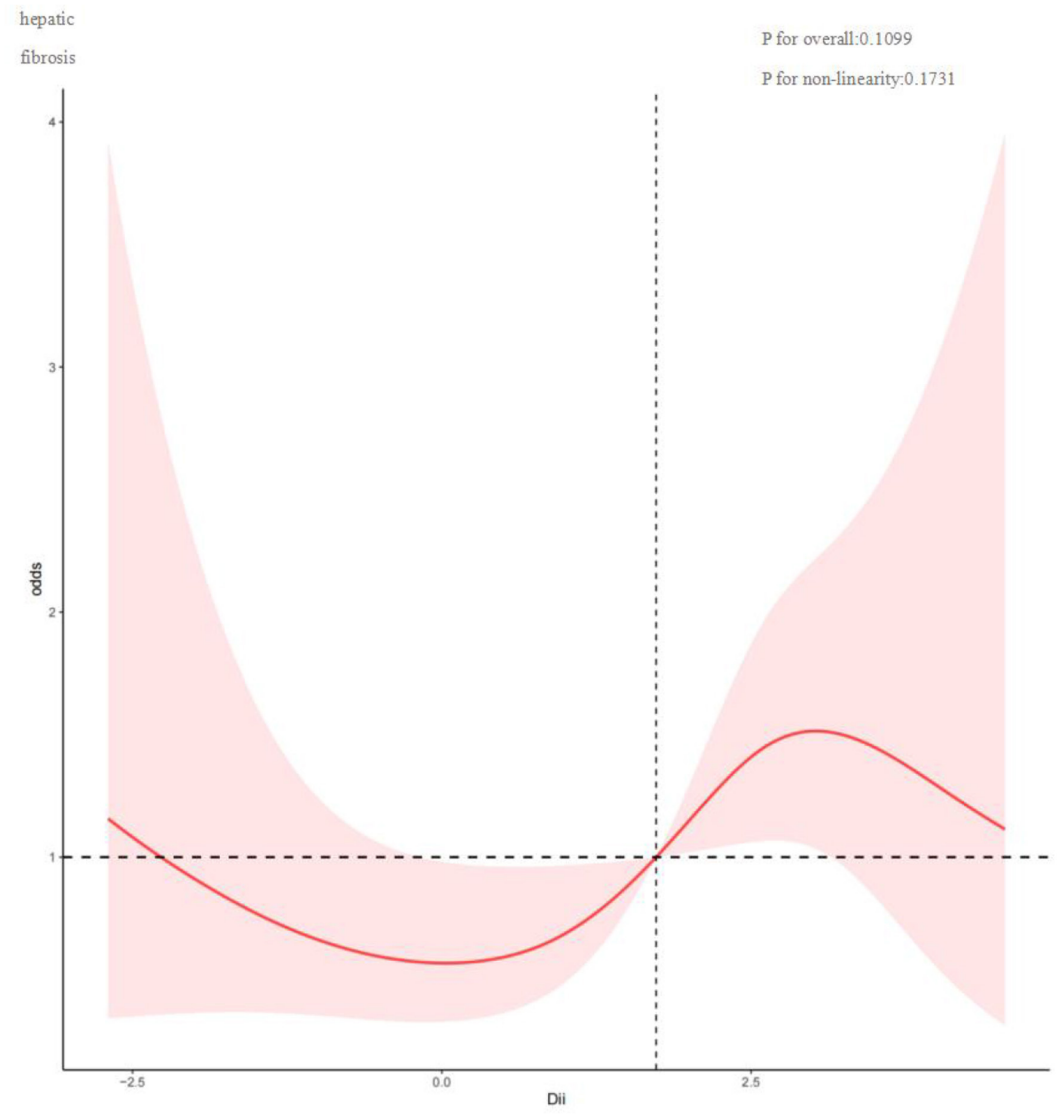
Restricted cubic spline curve (RCS) plot of the relationship between DII, and liver fibrosis.

To investigate potential nonlinear relationships between the Dietary Inflammatory Index (DII) and hepatic outcomes such as FLI and fibrosis among MASLD patients, restricted cubic spline analysis was conducted. As shown in Figure 2, DII and FLI exhibit a nonlinear correlation (*P* for nonlinearity = 0.0003), with inflection points at −0.075, 1.746, and 3.277. Overall, FLI increased with rising DII. Figure 3 depicts a nonlinear association between DII and liver fibrosis (*P* for nonlinearity = 0.1731), with two inflection points at 0.034 and 3.022.

### 3.5 Causal mediation analysis

To assess the potential mediating role of systemic immune-inflammation index (SII) in the association between dietary inflammatory index (DII) and metabolic dysfunction-associated steatotic liver disease (MASLD), we performed mediation analyses. However, these analyses did not reveal statistically significant mediation effects (Tables 7, 8).

**Table 7:** Mediation analysis of SII in the association between DII and FlI.

**Effect decomposition**	**β**	**95% CI lower**	**95% CI upper**	***P***
ACME	−0.029	−0.121	0.017	0.254
ADE	0.007	−0.829	0.754	0.970
Prop. Mediated	1.312	−1.614	1.042	0.946

**Table 8:** Mediation analysis of SII in the association between DII and liver fibrosis.

**Effect decomposition**	**β**	**95% CI lower**	**95% CI upper**	***P***
ACME	−0.001	−0.002	0.001	0.364
ADE	0.002	−0.013	0.017	0.760
Prop. Mediated	−0.227	−1.325	0.881	0.900

## 4 Discussion

This study evaluated the associations between the Dietary Inflammatory Index (DII), liver fibrosis, and the Fatty Liver Index (FLI) in individuals diagnosed with Metabolic Dysfunction-Associated Steatotic Liver Disease (MASLD), while also exploring the potential mediating role of the Systemic Immune-Inflammation Index (SII). The findings revealed no significant relationship between DII and either liver fibrosis risk or FLI among patients with established MASLD. Furthermore, mediation analysis based on SII did not support an indirect effect of diet-induced inflammation on hepatic disease progression through systemic inflammation. These null results suggest that, within the MASLD population, dietary inflammatory potential may not be directly linked to the extent of liver fibrosis or steatosis. Collectively, these findings highlight the need for a more nuanced and comprehensive exploration of the complex interplay between dietary factors, systemic inflammation, and liver-specific pathological changes in MASLD. This study enhances current knowledge of the interplay between dietary patterns, inflammation, and liver-related outcomes, and may provide important implications for designing individualized nutritional approaches and inflammation-focused therapeutic strategies in clinical practice.

Evidence from prior research indicates that higher DII scores are significantly associated with increased prevalence of metabolic syndrome and hepatic steatosis in the general population (10, 11, 39–42). However, our findings suggest that DII has limited predictive value for disease progression in individuals with MASLD, indicating that it may lack sufficient sensitivity in this context. This discrepancy highlights a potential disconnect between systemic inflammatory indicators and organ-specific pathology. As a tool designed to capture systemic inflammatory potential, the DII may not adequately reflect localized hepatic inflammatory micro environmental changes. Similarly, conventional inflammatory biomarkers such as C-reactive protein (CRP), while associated with MASLD onset, have also shown limited accuracy in predicting critical pathological processes such as fibrosis progression (43). Moreover, the DII is inherently constrained by its static nature and reliance on short-term dietary recall, making it ill-suited to capture the cumulative effects of chronic inflammation on liver health over time (44). This temporal mismatch between exposure assessment and disease evolution may further weaken its prognostic utility. Together, these findings underscore the limitations of systemic inflammation-based indices in assessing organ-specific diseases and point to the need for identifying more precise and tissue-specific biomarkers to improve MASLD risk stratification and disease monitoring.

More importantly, the “black-box” nature of DII calculation may obscure the differential effects of specific nutrients. This lack of mechanistic granularity—bridging macro-level indices with micro-level biological processes—limits its utility to statistical associations rather than predictive insights grounded in biological plausibility (41, 45). As an aggregate measure of dietary inflammation, the DII fails to delineate the distinct mechanisms through which individual dietary components influence hepatic pathophysiology. For example, n-3 polyunsaturated fatty acids—such as EPA and DHA—may alleviate inflammatory responses by inhibiting the NF-κB pathway, restraining hepatic stellate cell (HSC) activation, and reducing oxidative stress, thereby contributing to the deceleration of hepatic fibrogenesis (46, 47). In contrast, fructose bypasses insulin regulation and is rapidly metabolized in the liver into triose phosphates, lactate, and acetyl-CoA, directly promoting *de novo* lipogenesis and fibrogenesis (48, 49). Saturated fatty acids—for instance, palmitic acid—can trigger endoplasmic reticulum stress and impair mitochondrial function in hepatocytes, thereby promoting the overproduction of reactive oxygen species (ROS). The resultant oxidative stress not only damages hepatocytes but also activates adjacent HSCs, promoting their transdifferentiation into a profibrotic phenotype, thus accelerating the progression of nonalcoholic steatohepatitis (NASH) (50–52). This heterogeneity in nutrient-specific effects underscores the need to move beyond generalized inflammatory scores and toward mechanistic dissection of diet–liver interactions at the molecular level. Such insights are essential for developing targeted and effective nutritional interventions for MASLD.

Emerging evidence indicates that patients with MASLD exhibit a unique metabolic pathophysiological background, characterized by a triad of chronic low-grade inflammation, insulin resistance, and gut microbiota dysbiosis—collectively referred to as the “metabolic dysregulation triad” (53, 54). This distinct foundation gives rise to a phenomenon known as the “inflammatory threshold saturation,” wherein, once a baseline inflammatory plateau is reached, the marginal impact of dietary inflammatory burden is markedly diminished, reflecting a biological “ceiling effect.” A closer examination of the underlying mechanisms reveals that MASLD progression involves a dynamic shift in pathogenic drivers, following a temporally phased pattern. In the early stages, the Dietary Inflammatory Index (DII) primarily influences hepatic inflammation via the gut-liver axis, driven by dysbiosis-induced increases in portal vein lipopolysaccharide (LPS) (55). However, as the disease advances, hepatocyte lipotoxicity and hepatic stellate cell activation—induced by accumulated free fatty acids—gradually supersede inflammation as the dominant pathological mechanism (56). This transition from “exogenous inflammatory stimulation” to “endogenous metabolic derangement” parallels the natural course of type 2 diabetes, where early insulin resistance progresses to β-cell dysfunction, and ultimately culminates in β-cell apoptosis, dedifferentiation, and functional exhaustion (57, 58). In MASLD, this manifests as a gradual shift from inflammation-driven injury to mechanisms more directly rooted in metabolic toxicity and programmed cell death. Animal model data further corroborate this progression pattern. In high-fat diet-induced MASLD models, the contribution of gut-derived lipopolysaccharide (LPS) to hepatic inflammation shows a marked decline—from approximately 60% in early disease stages to around 20% in advanced phases (59–61). Concurrently, metabolic disturbances such as reduced secretion of adiponectin from visceral adipose tissue may offer greater explanatory power for advanced fibrosis than dietary inflammatory factors alone (62). Notably, sustained exposure to high-DII diets may induce adaptive shifts in the gut microbiota, thereby attenuating their pro-inflammatory potential over time (22). Moreover, systemic inflammation as reflected by the Systemic Immune-Inflammation Index (SII) may not effectively trigger liver fibrosis-specific signaling cascades. Collectively, these findings underscore a critical pathophysiological inflection point—transitioning from an intervention-responsive disease state to one in which therapeutic windows begin to close.

This study is subject to certain limitations. Most notably, the cross-sectional design precludes any inference of a causal relationship between the Dietary Inflammatory Index (DII) and the onset or progression of MASLD and hepatic fibrosis. Second, FLI, as an indirect marker of hepatic steatosis, may not fully capture actual liver fat deposition. Thirdly, the analysis was restricted to data from the 2017–2020 NHANES cycle due to the unavailability of complete information in other survey periods, which may constrain the temporal applicability of the results. Additionally, as NHANES exclusively surveys individuals residing in the United States, It remains uncertain whether these findings are generalizable to populations in different geographic regions or cultural contexts. Although the NHANES database provides biochemical indicators and questionnaire data on chronic conditions such as hypertension, diabetes, and hyperlipidemia, it lacks key variables related to medication dosage, treatment adherence, and duration of therapy. Therefore, we were unable to accurately distinguish between individuals with well-controlled and poorly controlled disease status. Moreover, due to a relatively small number of fibrosis-positive cases after database screening, the statistical power of some analyses may be limited. Nevertheless, we made every effort to enhance the reliability of our results by employing rigorous statistical methods, adjusting for major confounding factors, and conducting appropriate sensitivity and subgroup analyses.

These findings offer valuable insights with potential relevance to clinical decision-making and patient management. First, systemic inflammatory indices exhibit limited predictive value for MASLD, underscoring the need to develop organ-specific biomarker panels. Subsequent research should employ more detailed biomarker assessments to clarify the underlying biological mechanisms linking diet-induced inflammation with disease progression. Second, given the stage-dependent differences in MASLD pathophysiology, the timing of intervention is critical. Longitudinal cohort studies are warranted to examine the influence of the Dietary Inflammatory Index (DII) across different stages of the disease, with comprehensive integration of metabolic parameters, hepatic function indicators, and lifestyle-related variables. Third, nutritional interventions should be tailored to the dominant mechanisms at each disease stage—prioritizing enhancement of gut barrier function in the early phase and focusing on mitochondrial support during disease progression. Notably, the complex and potentially delayed association between DII and MASLD merits further investigation, as its long-term impact may only become evident through extended follow-up. Future research should pursue a multidimensional approach: leveraging animal models and organoid systems to investigate nutrient-driven hepatocyte–immune interactions; conducting multicenter longitudinal studies that integrate multi-omics and imaging data to construct stage-specific predictive models; and developing AI-driven nutritional platforms that synthesize dietary, metabolic, and genetic data to enable precision-based dietary interventions. Collectively, these insights provide a theoretical foundation for the precise prevention and management of MASLD and outline future directions, including the implementation of targeted interventions during critical therapeutic windows and the integration of multidimensional datasets to optimize clinical strategies.

## 5 Conclusion

Among individuals with MASLD in the United States, no meaningful statistical relationship was identified between DII scores and the risk of liver fibrosis or increased FLI. Despite established associations between DII and multiple chronic diseases, evidence regarding its role in hepatic fibrosis and FLI among the MASLD population remains limited and requires further elucidation. Notably, no direct mediating effect of SII was found between DII, FLI, and liver fibrosis.

These findings suggest that DII may lack specificity in capturing the intermittent or heterogeneous progression patterns of MASLD. This study provides new evidence for related fields, emphasizing that dietary factors should be carefully considered in the clinical evaluation of disease progression in patients with MASLD, and that the potential relationship between diet and liver disease progression warrants further investigation.

## Data Availability

The datasets presented in this study can be found in online repositories. The names of the repository/repositories and accession number(s) can be found in the article/supplementary material.
